# Antioxidative potential of *Lactobacillus* sp. in ameliorating D-galactose-induced aging

**DOI:** 10.1007/s00253-022-12041-7

**Published:** 2022-07-04

**Authors:** Harsh Kumar, Kanchan Bhardwaj, Marian Valko, Suliman Y. Alomar, Saleh H. Alwasel, Natália Cruz-Martins, Daljeet Singh Dhanjal, Reena Singh, Kamil Kuča, Rachna Verma, Dinesh Kumar

**Affiliations:** 1 School of Bioengineering & Food Technology, Shoolini University of Biotechnology and Management Sciences, Solan, 173229 India; 2 School of Biological and Environmental Sciences, Shoolini University of Biotechnology and Management Sciences, Solan, 173229 India; 3 Faculty of Chemical and Food Technology, Slovak University of Technology, 81237 Bratislava, Slovakia; 4 Zoology Department, King Saud University, College of Science, Riyadh, 11451 Saudi Arabia; 5 Faculty of Medicine, University of Porto, 4200-319 Porto, Portugal; 6 Institute for Research and Innovation in Health (i3S), University of Porto, 4200-135 Porto, Portugal; 7 Institute of Research and Advanced, Training in Health Sciences and Technologies (CESPU), Rua Central de Gandra, 1317, 4585-116 Gandra PRD, Portugal; 8 TOXRUN – Toxicology Research Unit, University Institute of Health Sciences, CESPU, 4585-116 Gandra, CRL Portugal; 9 School of Bioengineering and Biosciences, Lovely Professional University, Phagwara, Punjab 144411 India; 10 Department of Chemistry, Faculty of Science, University of Hradec Kralove, 50003 Hradec Kralove, Czech Republic; 11 Biomedical Research Center, University Hospital Hradec Kralove, Hradec Kralove, Czech Republic

**Keywords:** Aging, Antioxidant, D-galactose, Metabolites, Oxidative stress, *Lactobacillus*

## Abstract

**Abstract:**

Aging is a progressive, unalterable physiological degradation process of living organisms, which leads to deterioration of biological function and eventually to senescence. The most prevalent factor responsible for aging is the accumulation of damages resulting from oxidative stress and dysbiosis. D-galactose-induced aging has become a hot topic, and extensive research is being conducted in this area. Published literature has reported that the continuous administration of D-galactose leads to the deterioration of motor and cognitive skills, resembling symptoms of aging. Hence, this procedure is employed as a model for accelerated aging. This review aims to emphasize the effect of D-galactose on various bodily organs and underline the role of the *Lactobacillus* sp. in the aging process, along with its anti-oxidative potential. A critical consideration to the literature describing animal models that have used the *Lactobacillus* sp. in amending D-galactose-induced aging is also given.

**Key points:**

*• D-Galactose induces the aging process via decreasing the respiratory chain enzyme activity as well as ATP synthesis, mitochondrial dysfunction, and increased ROS production.*

*• D-Galactose induced aging primarily affects the brain, heart, lung, liver, kidney, and skin.*

*• The anti-oxidative potential of Lactobacillus sp. in improving D-galactose-induced aging in animal models via direct feeding and feeding of Lactobacillus-fermented food.*

## Introduction

Aging is a necessary stage of the lifecycle process and occurs naturally as the body ages. As an organism matures, a regular, genetically influenced, irretrievable decline in morphological formation and physiological function of the entire body occurs (Höhn et al. 2017; Klimova et al. 2018). Globally, the percentage of older people (of 60 years age or above) is increasing, and it is expected to increase by two-fold in 2050 (Azman and Zakaria 2019). Currently proposed mechanisms to elucidate the aging process include the (1) mitochondrial theory, (2) the free radical theory, (3) the immunological theory of aging, (4) the theory of damage to biofilms, and (5) the endocrine theory of aging (Larsson et al. 2019; Dhanjal et al. 2020). During the aging process, organisms undergo corresponding changes to their cells, molecules, tissues, and organs, and many diseases may occur in humans, like osteoporosis, cardiovascular, hypertension, cerebrovascular, and diabetes, with a gradual impact on the quality of life of the affected individuals (Booth and Brunet 2016).

Presently, because of simple application and maximum survival rate, a mimetic model of the aging of animals was preferred over using animals that had aged naturally (Azman and Zakaria 2021). D-galactose (aldohexose) is a reducing sugar, which is found inside the body and in several foodstuffs, including dairy products, like yogurt, cheese, milk, and butter, and in various fruits, like kiwi, plums, chestnuts and cherries, herbs, and vegetables (Azman and Zakaria 2021). On average, for healthy humans, the highest daily D-galactose-recommended dose is 50 g, from where after ingestion, it is further metabolized and removed in 8 h from the body (Morava 2014). D-galactose is converted into glucose by two enzymes, uridylyltransferase and galactokinase, which get involved in the glycolysis pathway or are kept in reserve in adipose tissue, muscles, etc. as glycogen (Coelho et al. 2015). In more significant amounts, these react with amino acids and free amines to form advanced glycation end products (AGEs), causing the progression with the development of several hepatic diseases (Azman and Zakaria 2019).

**Fig. 1:**
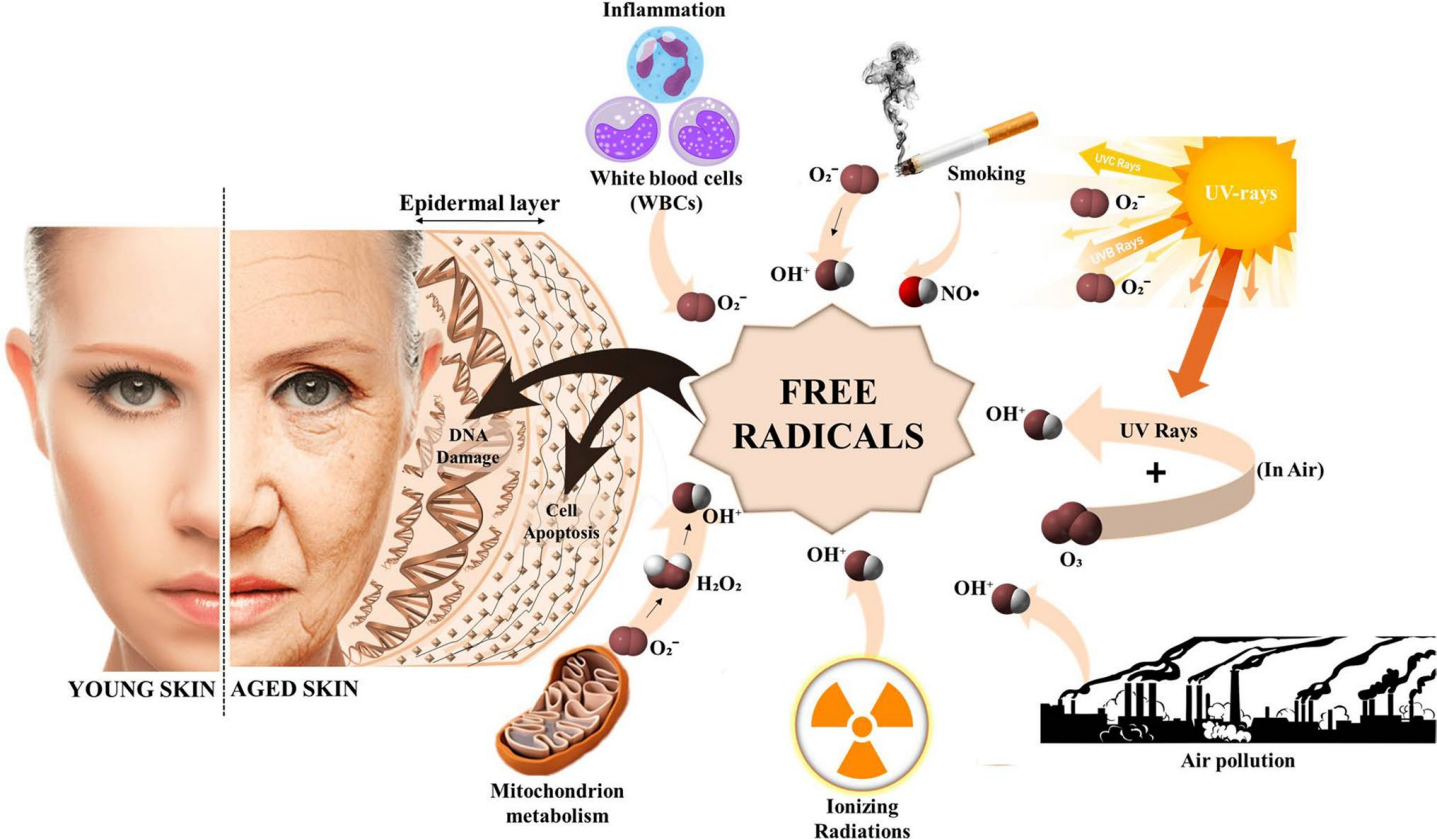
Illustration of different factors responsible for free radical generation resulting in aging

Additionally, at maximum concentrations, galactose oxidase catalyzes the oxidation of D-galactose into hydrogen peroxide, leading to the formation of reactive oxygen species (ROS). Aldose reductase converts D-galactose to galactitol, which is not further metabolized and increasingly becomes stored in cells leading to an accumulated free radical formation that may disturb redox balance and normal osmotic pressure (Yanar et al. 2011). Together, ROS, AGEs, osmotic stress, and redox imbalance formation may ultimately cause aging in organisms (Fig. 1). Although it is impossible to avert aging, various methods have been developed to delay the process (Zhou et al. 2021a). Thus, when *Lactobacillus* was employed in aging models during animal studies, significant protection was stated (Setbo et al. 2019; Ishaq et al. 2021). *Lactobacilli*, such as *Lactobacillus casei*, *Lactobacillus acidophilus*, *Lactobacillus rhamnosus*, *Lactobacillus paracasei*, *Lactobacillus delbrueckii* subsp. *bulgaricus*, *Lactobacillus johnsonii*, *Lactobacillus brevis*, *Lactobacillus fermentum*, and *Lactobacillus plantarum*, are frequently used as probiotic products (Fijan 2014; Kumar and Kumar 2015). The key benefits of probiotic species to host health include the production of neurotransmitters, immunomodulation, and improvements in barrier function (Ishaq et al. 2021). Moreover, *Lactobacilli* influence the hosts microbiota, gut-brain axis, and even cellular components (Ishaq et al. 2021). Lately, a study found that the intragastric feeding of heat-killed *L. paracasei* PS23 to aging mice model amends age-related muscle atrophy and regulates ghrelin levels (Cheng et al. 2021). Ghrelin is a vital gut hormone with pleiotropic effects, which controls nutrient sensing, meal initiation, and hunger. Moreover, it indirectly improves muscle mass by stimulating the insulin-like growth factor 1 (IGF1) pathway in mice with cachexia. Thus, these changes overall improve muscles’ functioning during aging (Cheng et al. 2021).

In this sense, this review aims to provide information regarding D-galactose-induced aging on several different bodily organs. A critical overview of the impact of the *Lactobacillus* sp. and its anti-oxidative potential is also provided, accompanied by the application of the *Lactobacillus* genus for ameliorating the D-galactose-induced aging in living organisms. Thus, this could serve as an evident report for food industrialist as well as researchers to develop functional food using *Lactobacillus* sp. not only because of their ability to serve as probiotic foods, but also as a food which has anti-aging effect.

## D-galactose metabolism

**Fig. 2:**
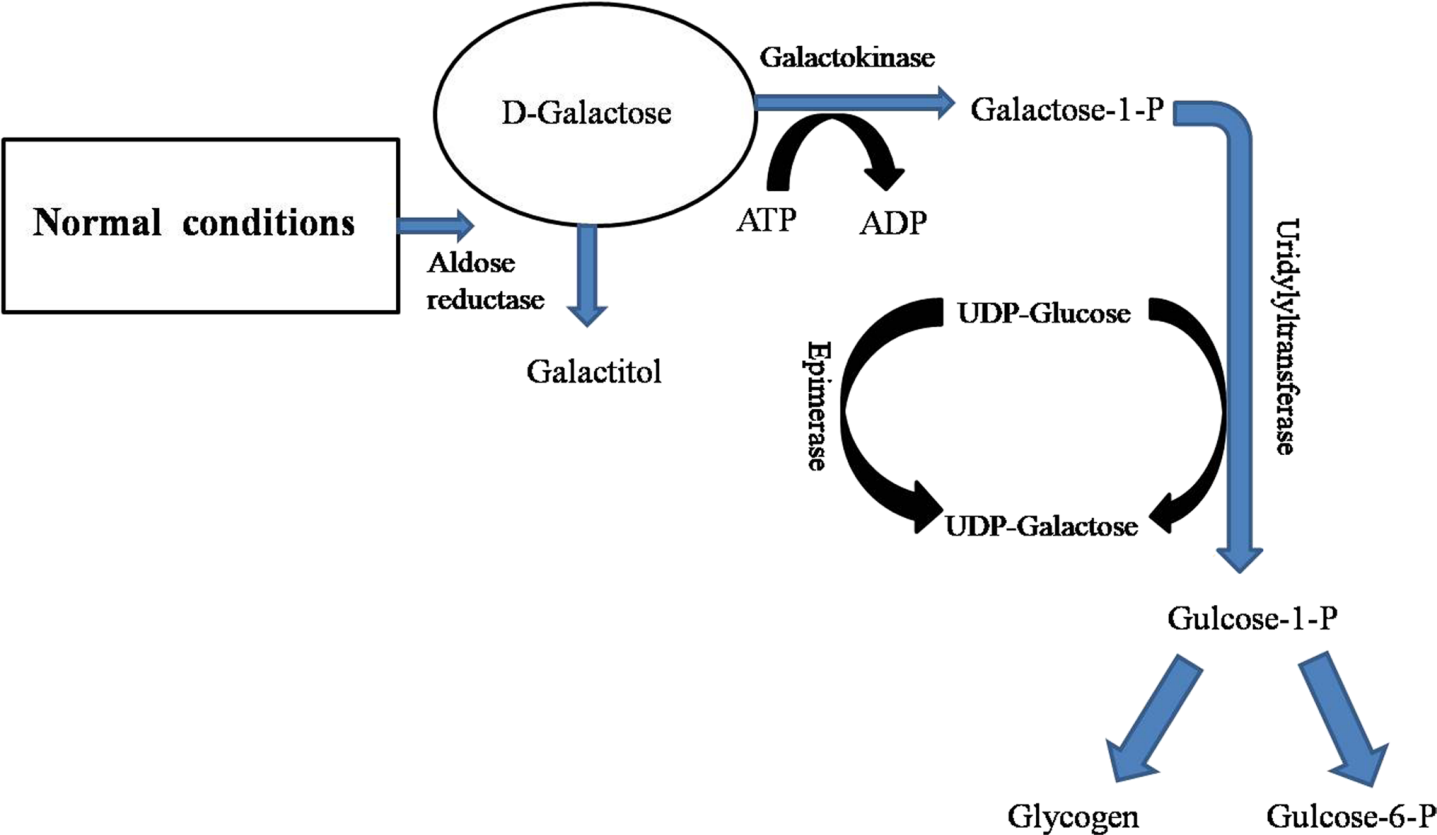
D-glactose metabolism pathway (Umbayev et al. 2020)

Galactose plays a significant role in different physiological processes; briefly, it is involved in the Schwann cells (PNS process) of the myelin sheath, the synthesis of heparin/heparan sulfates, and in the galactosylation of ceramide galactose (Coelho et al. 2015). In addition, it has been comprehended that it is endogenously formed in human cells, and an adult male of average weight 70 kg can synthesize almost 2 g of galactose per day (Berry et al. 1995, 1998). Generally, in endogenously produced galactose, the potential reaction mechanism is the hydrolysis of galactose in lysosomes containing glycolipids, glycoproteins, and proteoglycans (Berry et al. 1995, 1998). For instance, it has been demonstrated that galactose is metabolized primarily by the Leloir pathway (Fig. 2) (Holden et al. 2003). Initially, D-galactose is phosphorylated into galactose-1-phosphate (Gal-1-P) in the presence of galactokinase 1 (GALK1), and after D-galactose-1-phosphate, uridylyltransferase (GALT) uses uridine diphosphate to convert Gal-1-P to uridine diphosphate (UDP)-galactose. UDP-galactose is then converted into UDP-glucose by UDP-galactose 4′-epimerase (GALE). Subsequently, UDP-glucose reverses to the pathway, and galactose is further converted into UDP galactose and glucose-1-phosphate (G1P). Lastly, G1P is converted into glucose-6-phosphate via phosphoglucomutase. Galactose levels in the body can rise mainly by two means: (1) via raising consumption of galactose-rich foods and (2) by metabolic disorders of the Leloir pathway related to genetic mutations of enzymes (Lai et al. 2009). The D-galactose-induced aging effect on various body parts is discussed in the following sections.

## D-galactose-induced aging on few organs of the body

### Brain

**Fig. 3:**
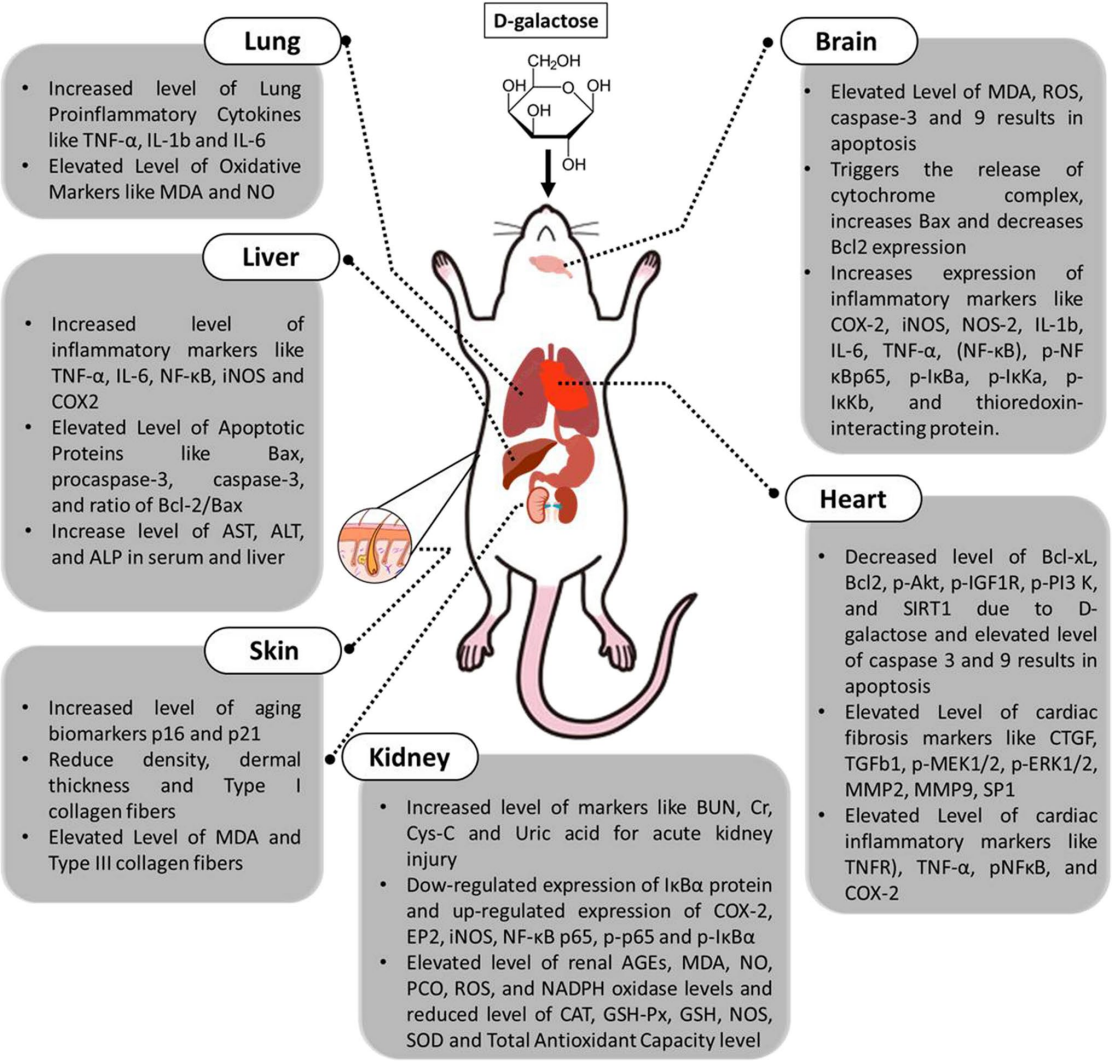
Schematic representation of effect of D-galactose on different body organs of animal model resulting in aging

It has been confirmed that the D-galactose-induced oxidative stress mechanism in the brain arises at a sub-cellular stage and exclusively in mitochondria (Shwe et al. 2018). Increasing levels of D-galactose are oxidized by galactose oxidase and converted into hydrogen peroxide (H_2_O_2)_, which leads to the reduction of superoxide dismutase (SOD) (Hsieh et al. 2009). Enhanced H_2_O_2_ reacts with the reduced form of iron (Fe) and is converted into hydroxyl radicals (OH). Therefore, OH and H_2_O_2_ are two forms of ROS, which, with several others, lead to lipid peroxidation of cell membranes and damage of redox homeostasis, causing neuronal dysfunction (Hsieh et al. 2009). Also, D-galactose binds to amino group compounds to form Schiff’s base products, which are unstable compounds that undergo numerous reactions to form more stable compounds over days, referred to as Amadori products (Shwe et al. 2018). The stable Amadori products change permanently to form AGE product compounds in months/years (Hsieh et al. 2009). When an AGE product combines with its receptor-advanced glycation end product (RAGE), the production of ROS and nicotinamide adenine dinucleotide phosphate (NADPH) oxidase increases, leading to cognitive dysfunction and neuron damage (Hsieh et al. 2009). However, D-galactose activates intrinsic and extrinsic apoptosis pathways (Shwe et al. 2018). At the mitochondria, the “death receptor” on the extrinsic pathway activates caspases enzyme through c-Jun-N-terminal kinase (JNK) and unites with the intrinsic apoptotic pathway (Shwe et al. 2018). One study revealed that D-galactose activates p-JNK, which increases the cytochrome complex (cyt c) levels, which further stimulates caspases-3 and -9 activation, and cleaves protein poly ADP ribose polymerase 1 (PARP-1) (Fig. 3) (Ali et al. 2015).

For monitoring D-galactose-induced models of aging, interleuki (IL-1β), cyclooxygenase 2 (COX-2), nitric oxide synthase-2 (NOS-2), nitric oxide synthase (iNOS), tumor necrosis factor alpha (TNF-α), IL-6, p-NF-κBp65, nuclear factor (NF)-κB, Txnip, p-IκBα, p-IKKβ, and p-IKKα have been analyzed, while recognized inflammatory markers. D-galactose is efficient in enhancing the inflammatory markers and allows neuro-inflammation through activation of the transcription factor NFκ-B via redox-sensitive signaling pathways and Ras, which results in memory damage (Shwe et al. 2018).

### Heart

Aging may cause coronary heart diseases related to ROS overproduction and oxidative stress. Treatment with D-galactose has been found to decrease levels of antioxidant enzymes, for example, catalase (CAT), SOD, glutathione peroxidase (GSH-Px), nitric oxide synthase (NOS), and also the total antioxidant capacity, which triggers oxidative stress within the animals’ heart tissues by raising concentrations of nitric oxide (NO) and malondialdehyde (MDA) (Li et al. 2005; Lei et al. 2016; Dehghani et al. 2018; Xu et al. 2019). Moreover, D-galactose was shown to raise the weights of the left ventricle weight and whole heart, a phenomenon that is associated with aging and hypertrophy (Chen et al. 2018). In addition, animals heart tissues administered with D-galactose have demonstrated a plumping of cardiac muscle fiber, a twisted shortening, and fuzzy structure and considerably widened the interval and congestion of myocardial interstitial capillary vessels (Lei et al. 2016).

### Lung

Aging primarily affects the lungs by increasing the size of alveoli and reducing elastic recoil, which may ease the closure of airways and increase the residual volume (Azman and Zakaria 2019). The treatment with D-galactose was responsible for altering lung elastic constitution (Sun et al. 2001). Furthermore, in beagle dogs, this also triggered lung injuries in the form of alveolar wall destruction and inflammatory infiltration, at a D-galactose dose of 50 mg/kg/day given till 90 days subcutaneously (Ji et al. 2017). Raised inflammatory markers COX2, iNOS, and NF-jB levels have also been found in beagle dog lungs compared to a control group of younger animals (Ji et al. 2017). The inflammatory responses due to D-galactose are related to oxidative stress. For example, in lung tissues, the treatment with D-galactose has been found to increase the levels of oxidative markers, e.g., MDA and NO, and to reduce the levels of antioxidant enzymes, e.g., CAT, NOS, GSH-Px, and SOD (Sun et al. 2001; Lei et al. 2016).

### Liver

Administration of excessive D-galactose causes galactitol and H_2_O_2_ accumulation resulting in the impairment of cell redox homeostasis (Azman and Zakaria 2021). In treated animals, the induction of D-galactose significantly reduced the enzyme levels, causing hepatic oxidative stress stimulation (Azman and Zakaria 2021). Moreover, D-galactose administration noticeably reduces the total antioxidant capacity (T-AOC) and the messenger RNA (mRNA) expression of NADPH quinone dehydrogenase 1 (NQO1) and heme oxygenase-1 (HO-1) in the liver, ultimately causing oxidative stress (Azman and Zakaria 2021).

Nuclear factor-erythroid 2 (Nrf2), which is considered to be an essential stress-response transcription factor, controls phase II detoxification enzymes, like GSH-Px, NQO1 HO-1, CAT, and SOD, as well as an array of antioxidants (He et al. 2020). Nrf2 is prominently related to Nrf2 mRNA reduction of protein, the aging process, and causes impaired signaling of Nrf2. In a study, it was found that the administration of D-galactose significantly reduced the liver Nrf2 relative gene expression level as compared to the control (Azman and Zakaria 2021). Keap1 effectively enhances the Nrf2 proteasomal degradation in the cell cytoplasm and significantly elevates the level of D-galactose (Saleh et al. 2019). Simultaneously, D-galactose causes oxidative stress in the hepatic organ via upregulating kelch-like ECH-associated protein 1 (Keap1). At the same time, the expression of Nrf2 is slowed down, leading to reduction of antioxidant or phase II enzyme expressions which eventually amends the liver damage and aging process.

Oxidative stress is mainly responsible for activating transcription factors, such as NF-κB pathway, whose main function is to regulate the expression of inflammatory genes responsible for inflammation in aging as well as its related diseases. It has been found that cytoplasmic protein IκBα is associated with NF-κB and slows down its physiological function. Proteasomes help to degrade IκBα and form p-IκBα through ubiquitination following phosphorylation. It has been shown that administration of D-galactose raised p-IκBα, p65 (NF-κB protein family member), and p-IκBα/IκBα ratios whereas reduces the p-p65/p65 ratio (Li et al. 2016; Jeong et al. 2017; Ji et al. 2017; Mo et al. 2017; Zeng et al. 2020). Also, D-galactose stimulated degradation of IκBα and activation of p65 nuclear translocation (Ruan et al. 2013). The p65 nuclear translocation process controls the expression of pro-inflammatory genes like COX2 and iNOS. Administration of D-galactose at a concentration of 200 mg per kg each day to male mice for 8 weeks subcutaneously resulted in a notable rise of COX2 and iNOS expression in the liver (Li et al. 2016; Mo et al., 2017). Likewise, in the liver, the subcutaneous administration of D-galactose for 90 days to a beagle dog at a dose of 50 mg/kg/day showed significant increase in COX2 and iNOS expression (Ji et al. 2017).

### Kidney

The physiological performance of the kidney reduces due to detrimental consequences of impaired redox homeostasis, in the advancement of aging. The proximal tubule, a segment of nephrons in the kidneys, contains an enormous amount of mitochondrial organelles, that is reliant upon oxidative phosphorylation, and is very susceptible to oxidant-induced DNA damage characterized by 8-hydroxy-2-deoxyguanosine (8-OHdG) inception (Hall and Unwin 2007). D-galactose treatment mimicked this aging process by instigating oxidative stress in the kidney tissues as it elevated renal AGEs, MDA, ROS, NO, protein carbonyl (PCO), and NADPH oxidase levels and diminishedtotal antioxidant capacity, and SOD, NOS, CAT, GSH-Px, and glutathione sulfhydryl (GSH) levels (Azman and Zakaria 2019). Oxidative stress may further trigger a variety of inflammatory transcription factors which may lead to the expression of inflammatory cytokines.

The administration of D-galactose upregulated the expressions of NF-κB p65, COX-2, iNOS, p-p65, p-IκBα, and EP2 and downregulated the IκBα protein expression (Azman and Zakaria 2019). The upregulation of the inflammatory transcription factors resulted in surged levels of inflammatory cytokines IL-6 and TNF-α, following D-galactose administration (Feng et al. 2016). Compared to the natural aging control, the D-galactose-treated rats also exhibited significantly higher lipid hydroperoxides (LHP) and 8-OHdG and significantly lower total thiol groups (T-SH) and protein thiol groups (P-SH) and higher LHP and 8-OHdG in the kidney tissues than the young control (Liu et al. 2010). D-galactose administration significantly increased the levels of uric acid and cystatin C (Cys-C), which is a marker for acute kidney injury, and blood urea nitrogen (BUN) and creatinine (Cr) (Fan et al. 2016; Azman and Zakaria 2019).

### Skin

In rats and mice, D-galactose administration caused a thinning of skin and deterioration of fur quality (Umbayev et al. 2020). In addition, the changes in hair color, the appearance of wrinkles and furrows in the skin, and the destruction of hair follicles were also noticed (Chen et al. 2017; Li et al. 2018; Sukoyan et al. 2018). Tian et al. (2011) found that when Kunming mice were administered with D-galactose, an accretion of subcutaneous fats occurred, and a reduction in the number of skin cell layers was observed. Wang et al. (2016) demonstrated that D-galactose administration reduced the skin tissue angiogenesis in mice. In D-galactose-treated animals, the skin moisture levels and skin aging biomarker were also reduced (Ye et al. 2014; Chen et al. 2016; Sukoyan et al. 2018). Furthermore, it was revealed that the animal’s skin, which was treated with D-galactose, showed raised expression of aging molecular biomarkers of p16 and p21 proteins, while cyclin D1 and sirtuin 1 (Sirt1) levels were decreased (Chen et al. 2019, 2016; Wang and Dreesen 2018). However, in male Wistar rats, administration of 125 mg/kg/day D-galactose for 6 weeks did not alter the skins water content to change (Liu et al. 2014).

Based on the above studies, special consideration was focused on collagen content and the quality of collagen fibers (Umbayev et al. 2020). It was found that the treatment of D-galactose in both rats and mice decreased the total skin collagen, while expression type I was downregulated, whereas the number of type III collagen was raised (Umbayev et al. 2020). The data collected through these studies showed that significant shortening and disordering of collagen fiber occurred in animals treated with D-galactose (Wang et al. 2013), which also caused dermal collagen fibers to be broken, slender, and sparse (Liu et al. 2014). In D-galactose-treated rat skin, collagen disorganization was accorded as they were found to be connected in the loosed manner (Chen et al. 2017). As per Ye et al. (2014), it has been observed that there is a loss of elastin in the skin of animals that were treated with D-galactose, while in addition, skin elastic fibers were lessened, scattered, and thinned in SD rats (Chen et al. 2013).

## *Lactobacillus* sp. cell architecture and properties

**Fig. 4:**
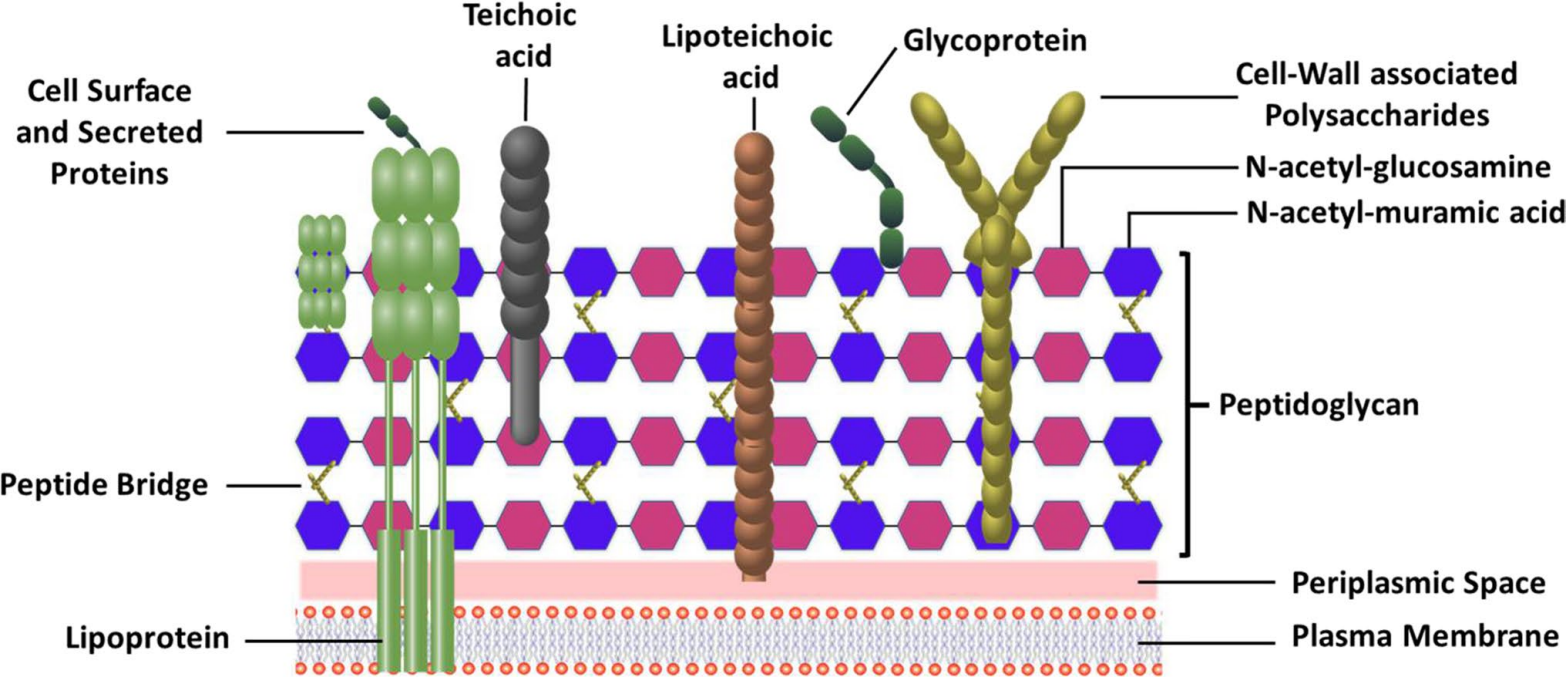
Diagrammatic illustration of the cell wall of *Lactobacillus* sp

A *Lactobacillus* cell wall is made up of a thick peptidoglycans (PG) multilayer, several β-1,4-linked N-acetylglucosamine units, and N-acetylmuramic disaccharide pentapeptide units cross-linked with a glycan chain (Fig. 4), where the prime composition of glycan strands and pentapeptides is characteristic for *Lactobacillus* strains (Kumar et al. 2021).

*L. plantarum* ATCC14917 PG, *L. casei*, and *L. johnsonii* JCM 2012 have been used to suppress IL-12 production while interacting with toll-like receptor 2 (TLR2), which further causes autoimmune diseases and inflammatory bowel disorders (Shida et al. 2009). Teichoic acid (TA) is the next major part of the *Lactobacillus* cell wall, accounting for its maximum dry weight (Teame et al. 2020). Moreover, the anionic polymer nature of TA allows the covalent linkage of the TA to peptidoglycans as wall teichoic acid (WTA) or it anchors to cytoplasmic membrane via lipid anchored lipoteichoic acid (LTA) (Teame et al. 2020). Several studies have revealed that TA from different species of *Lactobacillus* shows immunomodulatory effects (Kumar et al. 2021). For instance, IL-8 expression is affected by *L. plantarum* LTA induced via diacylated lipoprotein (Pam2CSK) and demonstrates the anti-inflammatory effect in epithelial cells of human intestine (Noh et al. 2015).

It has been revealed that some species of *Lactobacillus* exopolysaccharides (EPS) can control mucosal immune responses, which assists in the realization of direct health-promoting benefits. For example, *L. rhamnosus* RW-9595 M–produced purified EPS shows immuno-suppressive effects via exciting minimum amounts of TNF-α, IL-12, and IL-6, and high amounts of IL-10 in macrophages (Teame et al. 2020). Also, the *L. plantarum* BGCG11 strain shows immuno-suppressive activity by producing EPS anti-inflammatory effects (Kumar et al. 2021). Generally, EPS exerts immunoregulatory effects, although it also displays some anti-tumor properties. For instance, *L. plantarum* YW32 EPS exerted an anti-tumor response against colon cancer cells (HT-29) (in vitro) (Wang et al. 2015). Some *Lactobacillus* species, like *L. rhamnosus*, *L. helveticus*, *L. acidophilus*, and *L. plantarum*, have different surface proteins. For example, moonlight proteins, S-layer proteins, pili proteins, and LPXTG proteins have a considerable positive impact on various biological processes of the host (Liu et al. 2020). In particular, LPXTG proteins play a significant role in bacteria-host interactions by binding to epithelial and mucus cells (Jensen et al. 2014). It has also been stated that *Lactobacillus* strains obtained from natural dairy milk products, like *L. casei*, *L. rhamnosus*, and *L. paracasei* subsp. *paracasei* contains S-layer proteins which play a vital role in inhibiting the adhesion of *Shigella sonnei* to HT-29 cells (Teame et al. 2020). Furthermore, the pili binding to intestinal mucus leads to the persistence of the *Lactobacillus* strain in the GIT (Kumar et al. 2021).

## *Lactobacillus* sp. anti-oxidative activities

Kim et al. (2006) studied the shielding effect of oxidative damage from *L. gasseri* NLRI 312 in a Jurkat cell line of DNA and lipid from the cellular membrane. *L. gasseri* NLRI-312 displayed a defensive effect on the Jurkat cell lines against oxidative damage following supplementation and found that there is no effect on the production of MDA. *L. helveticus* cluster of differentiation-6 (CD6) helped to synthesize 5-methyl tetrahydrofolate (5-MeTHF), a derivative of folic acid, for anti-oxidative activity (Ahire et al. 2013). *L. helveticus* CD6 intracellular free extracts (ICFE) exerted anti-oxidative activity of ascorbate auto-oxidation with an inhibition rate of 27.5%. They demonstrated maximum Fe^+2^ metal ion chelation ability as compared to Cu^+2^. The intact cells hydroxyl radical scavenging and 2,2-diphenyl-1-picrylhydrazyl (DPPH) activities were 20.8% and 24.7%, respectively, as reported for their anti-oxidative potential. Yoon and Byun (2004) revealed that *L. casei* HY 2782 presents the highest GSH level among various tested probiotic strains. A significant positive correlation was observed between cellular GSH contents and antioxidant activity. *L. rhamnosus* GG superoxide anion radical scavenging capacity was more significant than accorded for various bacteria, e.g., *L. paracasei* YEC, *L. acidophilus* LA, *L. rhamnosus* Lc 705, *Escherichia coli*, and *Bifidobacterium* BB12 (Ahotupa et al. 1996). A few researchers have studied the *L. fermentum* FTL10BR and *L. fermentum* FTL2311 strains, as isolated from miang (traditional fermented tea leaves), which release particular substances that account for their antioxidant activity as expressed against trolox equivalent antioxidant capacity (TEAC) and free radical equivalent concentration (EC) values for scavenging and reducing mechanisms (Kullisaar et al. 2003).

*L. fermentum* displayed more excellent free radical scavenging potential than DPPH, hydroxyl, and superoxide radicals. At a 10^6^ and 10^9^ colony forming unit (CFU)/ml population, the scavenging activities for superoxide, hydroxyl, and DPPH were 12.86%, 7.35%, 64.26%, and 80.56%, 91.84%, 87.89%, respectively (Wang et al. 2009). Kapila and Vibha (2006) observed ICFE of *Lactobacillus* sp. 13 strains anti-oxidative activity using the linoleic acid peroxidation method and the microsome-thiobarbituric acid (MS-TBA) assay. The highest antioxidant capacity against oxidation inhibition was shown by *L. casei* ssp. *casei* 19 followed by *L. acidophilus* 14, *Lactobacillus* sp. L13, *L. casei* ssp. *casei* 63, *L. helveticus* 6, and *L. delbreuckii* ssp. *bulgaricus* 4, while the remaining strains showed less than 50% activity. In addition, the *L. casei* ssp. *casei* 19 strain showed peroxidation of linoleic acid (72.04%), *L. acidophilus* 14 (51.74%), and *Lactobacillus* sp. L13 (51.38%) while all other strains showed < 50% activity.

## *Lactobacillus* sp. role in ameliorating D-galactose-induced aging in animal models

### Direct feeding of Lactobacillus species

Ge et al. (2021) reported that intragastric administration of the *Lactobacillus plantarum* NJAU-01 at 10^9^ CFU/ml has the greatest T-AOC), SOD, CAT, and GSH-Px antioxidant enzymatic activities in the liver, heart, and serum of the treatment group of specific pathogen-free Kunming mice (SPF KM mice). *L. plantarum* NAJU-01 was able to promote the activity of antioxidant enzymes in mice and regulate ROS equilibrium to an average level. As an alternative mechanism, it might scavenge free radicals and work synergistically with CAT, GSH-Px, and SOD to lower oxidative stress. In male Kunming mice, it was observed that *L. fermentum* JX306, obtained from Chinese traditional fermented vegetable samples, significantly reduced MDA levels and enhanced the activity of GSH-Px, and total antioxygenic capacity (TOC) in the liver and kidney serum (Zhang et al. 2020). The strain showed a remarkable upregulation of the transcriptional level of antioxidant-related enzyme genes, such as thioredoxin reductase (TR3), glutathione peroxidase (Gpx1), peroxiredoxin1 (Prdx1), and glutathione reductase (Gsr) encoding genes in the liver (Table 1). *L. fermentum* HFY06, isolated from yogurt obtained from the yak in China, inhibited atrophy of kidneys, brain, spleen, and liver and enhanced serum MDA, GSH, SOD, and CAT levels in D-galactose-induced male Kunming mice (Li et al. 2021a, b). It has also been found that *L. fermentum* HFY06 is potent in alleviating D-galactose-induced aging in mice, owing to activation of the Nrf2 signaling pathway and enhancement of antioxidant enzymes and levels of downstream regulatory inflammatory factors.

**Table 1:** Experimental studies using animal models showing the anti-oxidative activities of *Lactobacillus* sp. against D-galactose-induced aging

Bacterial species	Bacterial dose/ duration	Animal model	D-galactose dose	Anti-aging benefits	Reference
*Lactobacillus fermentum* JX306	10^8^–10^10^ CFU/8 weeks	Kunming male mice	200 mg/Kg	↓ MDA ↑ GSH-Px, TOC, Prdx 1, Gsr, Gpx 1, and Tr3	Zhang et al. (2020)
*Lactobacillus plantarum* CQPC11, LP-CQPC11	1.0 × 10^9^ CFU/4 weeks	Specific pathogen-free Kunming mice (males and females)	120 mg/Kg	↓ MDA, and NO ↑ SOD, GSH-Px, and GSH ↑ *nNOS*, *eNOS*, *Cu/Zn-SOD*, *Mn-SOD*, *CAT*, *HO-1*, *Nrf2*, *γ-GCS*, and *NQO1*	Qian et al. (2018)
*Lactobacillus pentosus* var. *plantarum* (29)	1 × 10^10^ CFU/5 weeks	C57BL/6 J male mice	100 mg/Kg	↓ p16, p-p65, pFOXO3a, (COX)-2, and iNOS ↑ BDNF, DCX, and CREB Inhibited TNF-α, and arginase II Attenuated IL-10, arginase I, and CD206	Woo et al. (2014)
*Lactobacillus plantarum* CCFM10	10^9^ CFU/8 weeks	BALB/c male mice	1200 mg/Kg	↑ TOC, and GSH Restored the microbiota	Zhao et al. (2018)
*Lactobacillus helveticus* KLDS1.8701	NA/8 weeks	Specific pathogen-free BALB/c male mice	200 mg/Kg	↓ MDA ↑ Gpx, SOD, and TOC Manipulated the gut microbiota	Li et al. (2019)
*Lactobacillus paracasei* OFS 0291, *Lactobacillus helveticus* OFS 1515, *Lactobacillus fermentum* DR9	10 log CFU/6.4 weeks	Sprague Dawley male rats	600 mg/Kg	↓ MyoD, TNF-α, IL-6, IL-1β, and TRAP ↑ SOD, IGF-1, AMPK-α2	Hor et al. (2021)
*Lactobacillus plantarum* NJAU-01	10^9^ CFU/4 weeks	Specific pathogen-free Kunming female mice	500 mg/Kg	↓ MDA ↑TOC, SOD, GSH-Px, and CAT	Ge et al. (2021)
*Lactobacillus plantarum* AR501	10^10^ CFU/6 weeks	ICR male mice	10 g/Kg	↑ *Nrf2*, *Gsr*, *GST*, *GCL*, and *NQO1*	Lin et al. (2018)
*Lactobacillus mucosae* LMU 1001	NA/7 weeks	BALB/c male mice	50 mg/Kg	↑ *MT1*, *MT2*, *GPx1*, and *GPx2*	Yu et al. (2016)
*Lactobacillus plantarum* KSFY02	1 × 10^9^ CFU/6 weeks	Specific pathogen-free Kunming mice (males and females)	200 mg/Kg	↓ NO ↑ SOD, GSH-Px, and GSH	Zhao et al. (2019)
*Lactobacillus paracasei* ssp. *paracasei* YBJ01	5 × 10^10^ CFU/6 weeks	Kunming male mice	125 mg/Kg	Inhibited MDA ↑ SOD, GSH-Px, TOC, *CAT*, *Cu/Zn-SOD*, *Mn-SOD*	Suo et al. (2018)
*Lactobacillus fermentum* HFY06	1.0 × 10^10^ CFU/8 weeks	Kunming male mice	120 mg/Kg	↓ TNF-α, and IFN-γ ↑ *Nrf2*, *γ-GCS*, *NOS1*, *NOS3*, *SOD1*, *SOD2*, and *CAT*	Li et al. (2021a, b)
*Lactobacillus paracasei* M11-4	1 × 10^9^ CFU/8 weeks	Sprague Dawley male rats	200–600 mg/Kg	↑ *Trx*, and *GSH*	Yang et al. (2022)

*NA*, not applicable; *MDA*, malondialdehyde; *GSH-Px*, glutathione peroxidase; *TOC*, total antioxygenic capacity; *Prdx 1*, peroxiredoxin 1; *Gsr*, glutathione reductase; *Gpx 1*, glutathione peroxidase; *TR3*, thioredoxin reductase; *SOD*, superoxide dismutase; *GSH*, glutathione; *NO*, nitric oxide; *nNOS*, neuronal nitric oxide synthase; *eNOS*, endothelial nitric oxide synthase; *Cu/Zn-SOD*, cuprozinc-superoxide dismutase; *Mn-SOD*, manganese superoxide dismutase; *CAT*, catalase; *HO-1*, heme oxygenase; *Nrf2*, nuclear factor-erythroid 2 related factor 2; *γ-GCS*, γ-glutamylcysteine synthetase; *NQO1* NAD(P)H, dehydrogenase [quinine] 1; *BDNF*, brain-derived neurotrophic factor; *DCX*, hippocampal doublecortin; *CREB*, cAMP response element-binding protein; *iNOS* inducible nitric oxide synthase; *TNF-α*, tumor necrosis factor-α; *IL-*, nterleukin-10; *CD206*, cluster of differentiation 206; *IL-6*, interleukin-6; *IL-1β*, interleukin 1 beta; *TRAP*, tartrate resistant acid phosphotase; *AMPK-α2*, AMP activate protein kinase α2; *GST*, glutathione S-transferase; *GCL*, glutamate-cysteine ligase; *NQO1*, NAD(P)H quinine oxidoreductase 1; *MT1*, metallothionein 1; *MT2*, metallothionein 2; *GPx1*, glutathione peroxidase 1; *GPx*-, glutathione peroxidase 2; *IFN-γ*, interferon-gamma; *Trx*, thioredoxin

The anti-aging mechanisms were estimated for *L. helveticus* OFS 1515, *L. paracasei* OFS 0291, and *L. fermentum* DR9 in three probiotic strains on tibia and gastrocnemius muscles of rats subjected to D-galactose-induced aging (Hor et al. 2021). Upon induction of senescence, the aged rats showed reduction in the expression of anti-oxidative genes like SOD and CAT in both muscle and bone compared to younger rats (*P* < *0.05).* The *L. fermentum* DR9 strain showed amended SOD expression in muscle and bone compared to aged rats (*P* < *0.05*). It was revealed that SOD upregulation by *L. fermentum* DR9 in both tibia and gastrocnemius muscle represents a good defense in the community of antioxidant enzymes. Bacterial protective effects on muscle occurred by muscle regeneration stimulation by promoting anti-oxidative genes in an AMP activate protein kinase α2 (AMPKα2)-dependent manner. Li et al. (2019) reported that supplementation by *L. helveticus* KLDS1.8701 produced pure EPS, which extensively regulates the oxidative status like declined organic index, liver oxidative stress, and injury in D-galactose-induced aged male specific-pathogen-free BALB/c mice. These results obscured the prospect that supplementation of EPS lessens oxidative stress in the liver by amending the gut microbiota composition and serves as a probable candidate to reduce oxidative damage. The reduction in relative abundances of *Faecalibacterium*, *Lachnospiraceae* uncultured, *Lactobacillus*, *Bifidobacterium*, *Butyricicoccus*, *Blautia*, *Alloprevotella*, and *Rikenella* was recorded in the D-gal group in contrast to the control group. Still, these genera have been reported for improving EPS supplementation, except *Faecalibacterium*, because of selective nature of butyrate producer and probiotics for restablishing beneficial gut microflora. Zhao et al. (2018) reported that in D-galactose-induced aged male BALB/c mice, the administration of *Lactobacillus plantarum* CCFM10 enhanced the GSH and TOC levels and lessened *Clostridiales* and increased the *Lactobacillus* in the gut.

### Feeding of Lactobacillus fermented foods

Zhou et al. (2021a) revealed that fermented soymilk from *Lactobacillus plantarum* HFY09 increased the peptides and free soybean isoflavones. In an in vivo study of mice, it was found that fermented soymilk from *L. plantarum* HFY09 improved the levels of NO, T-SOD, CAT, GSH-Px, MDA, and GSH in the brain tissues, liver, and serum. The upregulation in the expression of HO-1, NOS3, Nrf2, NOS1, SOD1, CAT, SOD2, glutamate cysteine (Gclm), and quinone oxido-reductase 1 (Nqol), whereas, downregulated the expression of NOS2 which was recorded in the spleen and liver of mice supplemented with fermented soymilk obtained from *L. plantarum* HFY09. Furthermore, the upregulated expression of SOD2, SOD1, CAT, matrix metalloproteinases 2 (TIMP2), GSH-Px, and matrix metalloproteinases 1 (TIMP1) and downregulated expression of matrix metalloproteinases 9 (MMP9) and matrix metalloproteinases 3 (MMP3) was recorded in the skin. Another team of researchers reported reduced aging in D-galactose-induced mice and proposed the involvement of Nrf2 signaling pathway activated via small molecule peptides and soy isoflavone aglycone. Zhou et al. (2021b) observed that fermented soymilk from *Lactobacillus fermentum* CQPC04 contained large amounts of isoflavones as well as peptides that are generally accountable for reducing aging in D-galactose-induced aging mice. In other reports, fermented soymilk from *Lactobacillus plantarum* HFY01 was demonstrated to protect the liver, spleen, and skin and has potency to decrease inflammation. Oxidative damage D-galactose treated Kunming female mice (Li et al. 2021a, b). Elsewhere, it was revealed that the component isoflavones, genistein, daidzein, glycitein, daidzin, and glycitin are responsible for anti-oxidative activities.

## Conclusion and future perspectives

Aging is a global problem that each individual confronts every day. Thus, exploring therapeutic approaches for regulating aging is extremely important. For the survival, the coordinated control and normal functioning of organs are essential, and each organ plays a critical role in the body’s overall functioning. D-galactose-induced mimetic aging is associated with apoptosis, mitochondrial dysfunction, and inflammation. The clinical outcomes often lead to cognitive impairment, cardiovascular disease, liver disorders, and skin issues. Different therapeutic approaches targeting oxidative stress markers, inflammation, and mitochondria have been developed to alleviate the aging induced by D-galactose. Recently, those scientific reports related to probiotics have revealed their importance in regulating the hosts immune system, cognition, and antioxidant system. It has also been found that dietary factors play a vital role in mortality and longevity.

Interestingly, supplementation with species of *Lactobacillus* sp. significantly affects the gut microbiota, yielding anti-aging properties and improving the individuals’ general health. Studies conducted to date have underlined the potential of *Lactobacillus* species in strengthening the resistance of cells, tissues, and organs, thus improving the outcome of aging and age-related issues. Therefore, being aging a natural and physiological process, from data presented here, the use of the *Lactobacillus* sp. may be a new window of opportunities for researchers to explore their potential for effectively ameliorating the aging process and its associated problems and to extend the lifespan of humans.

## Data Availability

The authors confirm that the datasets supporting the findings and conclusion of this study are availbe within the article.
